# When an Oral Lesion Uncovers Hyperparathyroidism: A Peripheral Brown Tumor Case

**DOI:** 10.1002/ccr3.72626

**Published:** 2026-04-28

**Authors:** Bruno Teixeira Gonçalves Rodrigues, Vanessa Freitas Pacheco, Livia Xavier Costa do Rego Moreira, Mariana Cartaxo Faustini, Nathália de Almeida Freire, Danilo Passeado Branco Ribeiro, Mônica Simões Israel

**Affiliations:** ^1^ Oral & Maxillofacial Surgery, Pedro Ernesto University Hospital State University of Rio de Janeiro Rio de Janeiro Brazil; ^2^ Department of Oral Medicine Faculdade São Leopoldo Mandic Rio de Janeiro Brazil; ^3^ Oral Medicine, Department of Diagnosis and Therapeutics, Dental School Rio de Janeiro State University Rio de Janeiro Brazil

**Keywords:** giant cell tumors, hyperparathyroidism, oral, oral medicine, pathology

## Abstract

Oral lesions may represent the first sign of an underlying systemic disease. Giant cell lesions in the oral cavity should prompt investigation for hyperparathyroidism, as early recognition of brown tumors allows appropriate systemic management and may lead to lesion regression after treatment of the endocrine disorder.

## 1 Case Presentation

**FIGURE 1:**
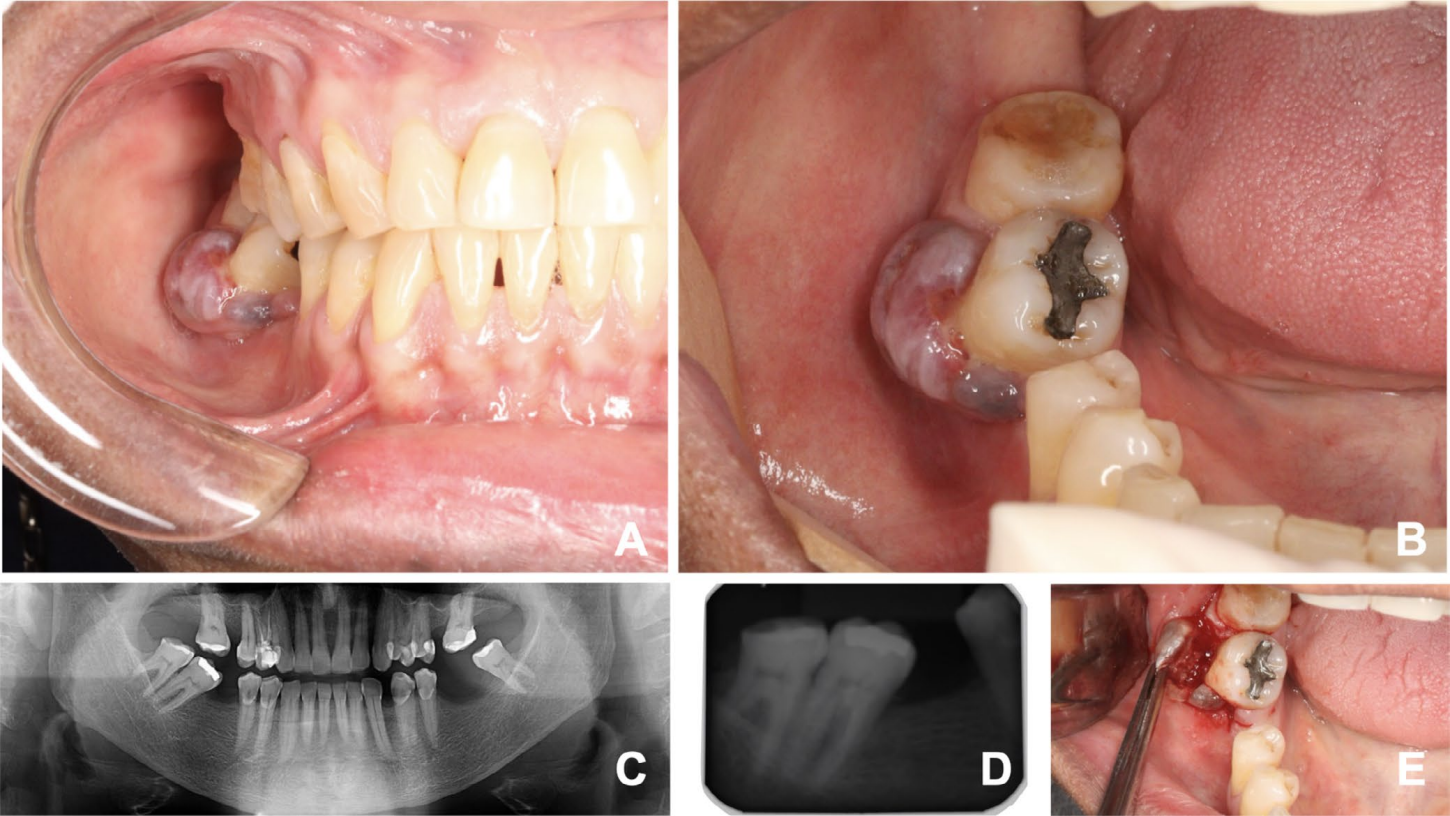
Clinical and radiographic features of a peripheral oral brown tumor. (A, B) A solitary, rounded, exophytic lesion with a bluish coloration located on the buccal aspect of the right mandibular second molar. (C, D) Panoramic and periapical radiographs showing no evidence of underlying bone involvement. (E) During the excisional biopsy, significant bleeding occurred, requiring additional hemostatic measures, including the use of local hemostatic agents.

A 40‐year‐old male patient presented with a 2‐month history of progressive swelling in the mandible. Medical history was unremarkable. Intraoral examination revealed a well‐circumscribed, bluish, exophytic lesion on the buccal aspect of the right mandibular second molar (Figure 1A,B). Radiographic imaging showed no underlying bone involvement, suggesting a peripheral lesion (Figure 1C,D).

**FIGURE 2:**
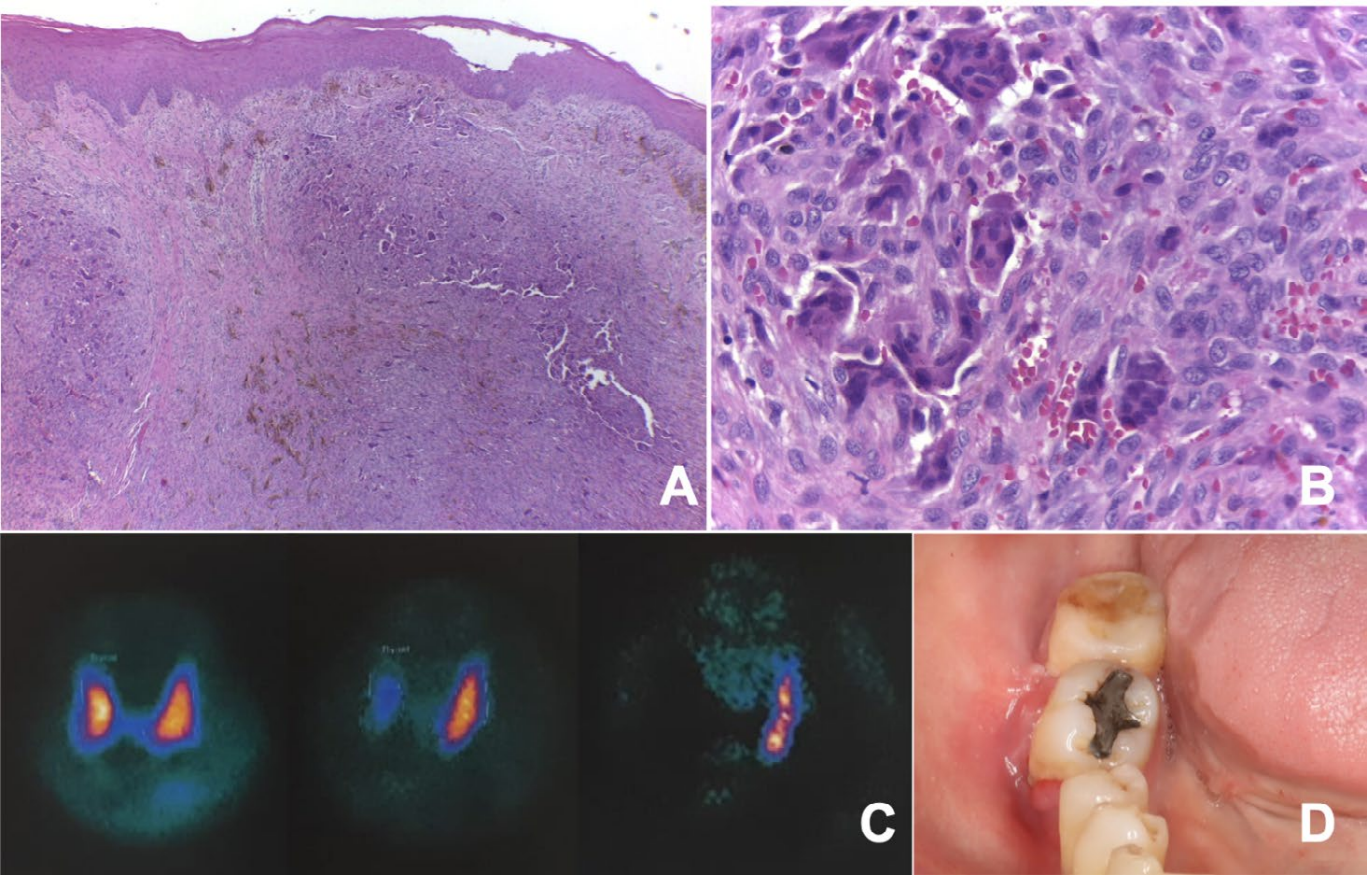
Histopathological, scintigraphy and follow‐up features of a peripheral oral brown tumor. (A) Proliferation of mononuclear ovoid and spindle‐shaped cells associated with multinucleated giant cells and areas containing hemosiderin deposits. (B) Multinucleated giant cells with focal hemorrhagic areas within the connective tissue stroma (H&E stain, original magnification ×400). (C) Parathyroid scintigraphy with 99mTc‐sestamibi associated with SPECT–CT demonstrating focal increased uptake in the left inferior region, consistent with a hyperfunctioning parathyroid gland (adenoma). The hybrid imaging allows precise anatomical localization, with persistent tracer retention on delayed images supporting the diagnosis. (D) Intraoral clinical image obtained 6 months after excisional biopsy, showing complete healing of the surgical site with no evidence of lesion recurrence.

Based on clinical findings, differential diagnoses included peripheral giant cell lesion and pyogenic granuloma. An excisional biopsy was performed, with notable intraoperative bleeding (Figure 1E). Histopathological analysis demonstrated a proliferation of spindle‐shaped mesenchymal cells within a fibrous stroma, with multinucleated giant cells and hemorrhagic areas (Figure 2A,B). Given these microscopic findings, the lesion was classified as a giant cell lesion, and additional laboratory investigations were requested to assess the possibility of a brown tumor (BT) associated with hyperparathyroidism (HPT).

Laboratory tests revealed elevated parathyroid hormone (PTH: 363 pg/mL) and alkaline phosphatase (337 U/L). Further endocrinological investigation demonstrated vitamin D deficiency (17.2 ng/mL), normal renal function (creatinine 0.79 mg/dL; eGFR > 90 mL/min/1.73 m^2^), hypercalciuria (486 mg/24 h), and hypercalcemia (11.4 mg/dL). Parathyroid scintigraphy identified a hyperfunctioning left inferior parathyroid gland (Figure 2C). Bone densitometry (DEXA) revealed osteoporosis.

Based on these findings, a diagnosis of primary HPT was established. The patient was initiated on vitamin D supplementation (2000 IU/day) and risedronate (150 mg/month) and referred for parathyroidectomy, which is currently pending. However, at the 6‐month follow‐up, no signs of peripheral BT recurrence were observed (Figure 2D).

## 2 Discussion

The oral cavity can provide important clues to underlying systemic diseases, as several conditions may present with oral manifestations [1, 2, 3]. In some cases, the first clinical sign of a systemic condition may appear in the mouth, as observed in this study featuring a peripheral BT associated with HPT [3].

HPT is classified according to its etiology. Primary HPT results from excessive secretion of PTH by one or more parathyroid glands, leading to hypercalcemia. Secondary HPT occurs as a compensatory response to persistent hypocalcemia, often related to vitamin D deficiency or metabolic disorders. Tertiary HPT typically develops in patients with chronic renal failure after prolonged secondary HPT, when the parathyroid glands become autonomous. Finally, a less common form, ectopic HPT, may occur when elevated PTH levels originate from nonparathyroid tissues, frequently associated with certain malignancies [1, 2].

BTs are non‐neoplastic giant cell lesions related to HPT, occurring in approximately 1.5%–4.5% of cases. Maxillofacial involvement is uncommon but shows a predominance in the mandible [3]. Radiographically, these lesions usually present as well‐defined osteolytic areas; however, exclusive soft tissue involvement is rare [1], as observed in the present case. Therefore, whenever a giant cell lesion is diagnosed, further investigation is necessary to rule out HPT.

Early recognition is essential because treatment must focus on the underlying endocrine disorder, which may require surgical and/or pharmacological management. Once HPT is controlled, lesion regression may occur and recurrence is uncommon. Consequently, dentists should integrate clinical, radiographic, histopathological, and laboratory findings and collaborate with medical specialists to achieve an accurate diagnosis and appropriate management.

## Author Contributions

**Vanessa Freitas Pacheco:** writing – original draft, investigation. **Nathália de Almeida Freire:** writing – original draft, investigation, writing – review and editing. **Mariana Cartaxo Faustini:** writing – original draft, investigation. **Livia Xavier Costa do Rego Moreira:** investigation, writing – original draft. **Mônica Simões Israel:** conceptualization, investigation, writing – original draft, writing – review and editing. **Danilo Passeado Branco Ribeiro:** investigation, conceptualization, writing – original draft, writing – review and editing. **Bruno Teixeira Gonçalves Rodrigues:** conceptualization, funding acquisition, writing – original draft, investigation, writing – review and editing.

## Funding

The authors have nothing to report.

## Ethics Statement

The authors have nothing to report.

## Consent

Written informed consent for the publication of this case, including all clinical details and any accompanying images, was obtained from the patient.

## Conflicts of Interest

The authors declare no conflicts of interest.

## Data Availability

Data sharing not applicable to this article as no datasets were generated or analysed during the current study.
